# Psychosocial impact of COVID-19 on cancer patients, survivors, and carers in Australia: a real-time assessment of cancer support services

**DOI:** 10.1007/s00520-021-06101-3

**Published:** 2021-03-11

**Authors:** Rhiannon Edge, Carolyn Mazariego, Zhicheng Li, Karen Canfell, Annie Miller, Bogda Koczwara, Joanne Shaw, Natalie Taylor

**Affiliations:** 1 Cancer Research Division, Cancer Council NSW, 153 Dowling Street, Sydney, NSW 2011 Australia; 2 The University of Sydney School of Public Health, University of Sydney, Sydney, Australia; 3 Cancer Information & Support Services, Cancer Council NSW, Sydney, Australia; 4 Flinders Medical Centre, Adelaide, SA Australia; 5 Flinders University, Adelaide, SA Australia; 6 Psycho-oncology Co-operative Research Group, School of Psychology, The University of Sydney, Sydney, Australia

**Keywords:** Coronavirus, Pandemic, Supportive care, Cancer survivorship

## Abstract

**Purpose:**

This study aimed to explore the psychosocial impacts of the coronavirus disease (COVID-19) pandemic on cancer patients, survivors, and carers in Australia.

**Methods:**

Using real-time insights from two Cancer Council NSW services—131120 Information and Support Line and Online Community (CCOC) forums—we assessed service demand trends, distress levels (using the distress thermometer), and content from 131120 calls and online posts between 01 December 2019 and 31 May 2020. Emergent themes were identified through an inductive conventional content analysis with 131120 call notes, followed by a deductive directed content analysis on CCOC posts.

**Results:**

In total, 688 COVID-19-related 131120 calls (*n* = 496) and online posts (*n* = 192) were analysed. Service demand peaked in March 2020 and self-reported distress peaked in May 2020 at an average of 8/10 [Mean = 7.5; SD = 0.9]. Five themes emerged from the qualitative analysis: psychological distress and fear of virus susceptibility, practical issues, cancer service disruptions, information needs, and carer Issues.

**Conclusions:**

The psychosocial impacts of COVID-19 on people affected by cancer are multifaceted and likely to have long-lasting consequences. Our findings drove the development of six recommendations across three domains of support, information, and access. Cancer patients, survivors, and carers already face stressful challenges dealing with a cancer diagnosis or survivorship. The added complexity of restrictions and uncertainty associated with the pandemic may compound this. It is important that healthcare providers are equipped to provide patient-centred care during and after this crisis. Our recommendations provide points of consideration to ensure care is tailored and patient oriented.

**Supplementary Information:**

The online version contains supplementary material available at 10.1007/s00520-021-06101-3.

## Introduction

The coronavirus disease (COVID-19) pandemic is a global health crisis of unparalleled magnitude. Since the initial outbreak in December 2019, 100 million confirmed cases have been recorded globally, with over 28,000 infections and 900 deaths in Australia as of January 2021 [1]. Emerging evidence has identified significant psychosocial impacts on a global scale [2, 3], with people experiencing fear and anxiety about the risks of COVID-19, uncertainty regarding the deteriorating global economy, and reduced social connectedness due to internationally mandated virus control measures such as social distancing, quarantine, and travel restrictions [4].

Although evidence is still emerging, cancer patients and survivors may be at higher risk of severe COVID-19 symptoms or fatal illness, potentially due to the immuno-suppressant effect of common oncological treatments such as chemotherapy, radiation, and immunotherapy [5, 6]. Additionally, as cancer incidence rates increase due to population ageing, this further elevates the risk of adverse COVID-19 outcomes [7]. These vulnerabilities and the reconfiguration of cancer services to minimise risks have driven public health guidance for cancer patients, survivors, and their carers to take extra precautions—including self-isolation and reduced physical contact, significantly altering social and peer support networks [8].

Both in Australia and internationally, there are growing concerns about the secondary effects of the pandemic on cancer incidence and mortality due to major service disruptions [9, 10]. Declining cancer incidence rates have been observed in several countries since the onset of COVID-19, with the Netherlands Cancer Registry Data presenting a 40% decline [11] and emerging data from the UK revealing marked reductions in GP referrals for suspected cancer of up to 60% [12]. In Australia, early indications from specialist oncology centres exhibit similar reductions in cancer referrals and recent data from the Australian Institute of Health and Welfare (AIHW) indicated there were 145,000 fewer mammograms performed between January to June 2020, when compared to January to June 2018 due to screening program disruptions [13, 14]. These unintended consequences may delay not only cancer diagnosis and treatment, but also the recognition and management of significant psychological morbidity.

Given the complex implications of COVID-19 on this vulnerable population, there is an urgent need to explore the real-time psychological, practical, and social impact of the pandemic, and to investigate specific unmet needs of cancer patients, survivors, and carers [15]. Two services provided by Cancer Council New South Wales (CCNSW) that have been pivotal in supporting people affected by cancer include the 131120 Information and Support Line (131120) and Cancer Council’s Online Community (CCOC)—a public online peer support forum. Each month, 131120 typically receives >800 calls and CCOC receives >10,000 visits from the Australian public, offering a wealth of unfeigned insight from the perspectives of cancer patients, survivors, and carers. Using emergent data from these two CCNSW services, this research aims to understand how COVID-19 has impacted the psychosocial wellbeing of cancer patients, survivors, and carers in Australia.

## Methods

### Conceptual design

This study utilised a mixed-methods descriptive approach, with a particular focus on qualitative content analyses. This methodology allows for natural inquiry of the phenomenon of interest, ultimately facilitating the discovery of themes without preconceived hypotheses [16].

### Sampling

Data were extracted from two sources: calls made to CCNSW’s 131120 service and online discussion posts on the CCOC forums. Calls to CCNSW’s 131120 service are incoming from New South Wales (NSW) and Australian Capital Territory (ACT). Consultants record information about the calls according to predetermined categories that are collected as part of a National Minimum Dataset (NMDS), including the following: caller demographics (e.g. age, gender), main reason for call (e.g. practical issues, general information), type of caller (e.g. patient, carer), and cancer stage and type. The NMDS also includes the distress thermometer (DT)—a validated measurement tool used for routine distress screening in cancer patients, utilising a self-reported 0 to 10 rating scale [17]. Throughout call interactions, consultants also log notes of the themes, outcomes, and recommendations. Call records are subsequently stored in a secure database at CCNSW, allowing for the extraction of aggregated descriptive data and keyword searches on the call notes. Online posts on the CCOC discussion forums are publicly available for viewing, although individuals must register for membership to post content.

### Procedure

131120 call notes and CCOC online posts were eligible for analysis if they occurred over a 6-month period between 01 December 2019 and 31 May 2020, during which time the pandemic unfolded globally and social restrictions were mandated by the national and jurisdictional governments. The study start date, commencing from 01 December 2019, was chosen to ensure all potential 131120 calls and CCOC online posts relating to the pandemic were captured, and for benchmarking service demand against data from early December and previous years. Records were extracted from the 131120 call database if the following terms were included in the call notes: ‘covid, corona, coronavirus, corono, coronovirus, pandemic, virus’. Adopting a consistent approach (Supplementary Table 3), CCOC online discussion posts were searched using the same terms and extracted for analysis. Calls were excluded if callers did not identify as cancer patients, survivors, or carers (e.g. healthcare professionals). Online posts were excluded if they were not specifically relevant to the COVID-19 pandemic.

### Data analysis

Descriptive data from the 131120 calls and CCOC forums were extracted and examined to assess trends in service demand (e.g. call volumes and pageviews) over the study period. Additionally, caller distress levels (using DT ratings) and other relevant health and demographic information were reported for sample characterisation and to monitor trends over time. Data from the study period (01 December 2019–31 May 2020) were also benchmarked against an equivalent period in the 2018/19 financial year (01 December 2018–31 May 2019) for comparison.

Two content analyses were then conducted on eligible calls and online posts using a qualitative descriptive approach. First, an inductive conventional content analysis was undertaken (RE and ZL) on the 131120 call data. Call notes were openly coded, added to coding matrices, grouped into higher order headings and associated sub-themes/categories, and prepared for the abstraction of major themes [18]. Themes identified from 131120 call data were then used to focus a secondary deductive directed content analysis on the CCOC online posts (CM). Line codes, major themes, and sub-themes identified from the call note analysis were used as initial coding categories that guided further iterations to the developing thematic framework [19]. To protect confidentiality, researchers only accessed de-identified data and restricted the use of 131120 verbatim quotes; illustrative 131120 quotes and publicly available CCOC quotes are presented to support emergent themes.

## Results

**Fig. 1:**
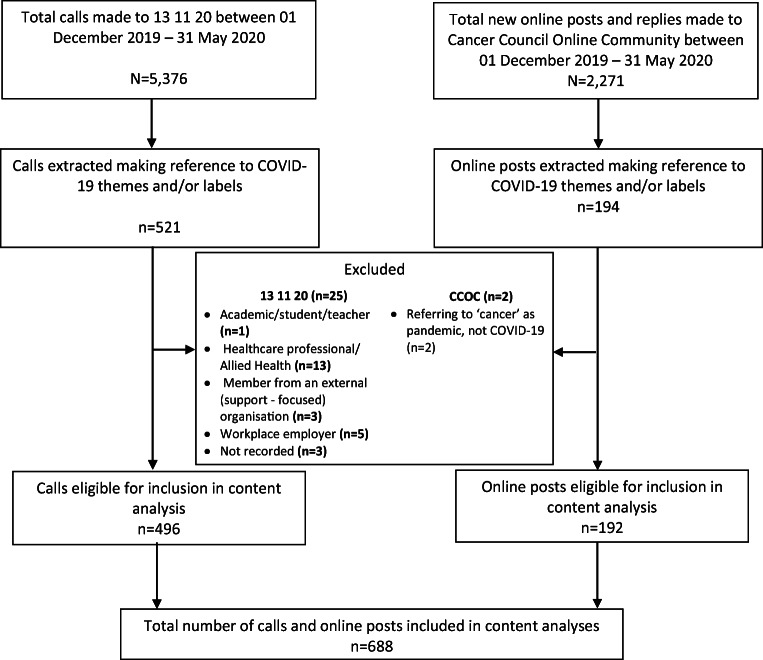
Flowchart of included data from calls to 13 11 20 and posts on Cancer Council’s Online Community (CCOC) for content analyses

The analysis included a total of 688 calls (*n* = 496) and online posts (*n* = 192) (Fig. 1). The majority of callers were aged 40–69 (40–49 [18%], 50–59 [19%], 60–69 [21%]), female (80%), had a cancer diagnosis (58%), and had either been diagnosed or were calling on behalf of someone with early stage localised disease (39%) (Table 1). Due to the anonymity of the CCOC service, specific demographic data for CCOC posters was not available.

**Table 1:** Demographic characteristics and descriptors of calls to Cancer Council NSW’s 13 11 20 service included in COVID-19-themed content analysis^*^

Category	*N* = 496	%*N*
Age range		
0–19	2	0%
20–29	13	3%
30–39	39	8%
40–49	87	18%
50–59	94	19%
60–69	101	21%
70–79	52	11%
80+	25	5%
Unknown	71	15%
Gender		
Female	397	80%
Male	98	20%
Type of caller		
Family/friends	176	35%
General public	33	7%
Someone with cancer	287	58%
Stage of cancer		
First diagnosis (localised)	191	39%
Second primary	3	1%
Metastasis/widespread/advanced	126	25%
Terminal stage	18	4%
Reoccurrence	10	2%
Stable disease	30	6%
Stage unknown/not applicable	114	23%

^*^These data are collected as part of the National Minimum Dataset (NMDS) that all Cancer Council’s in Australia are required to record

### Service demand

**Fig. 2:**
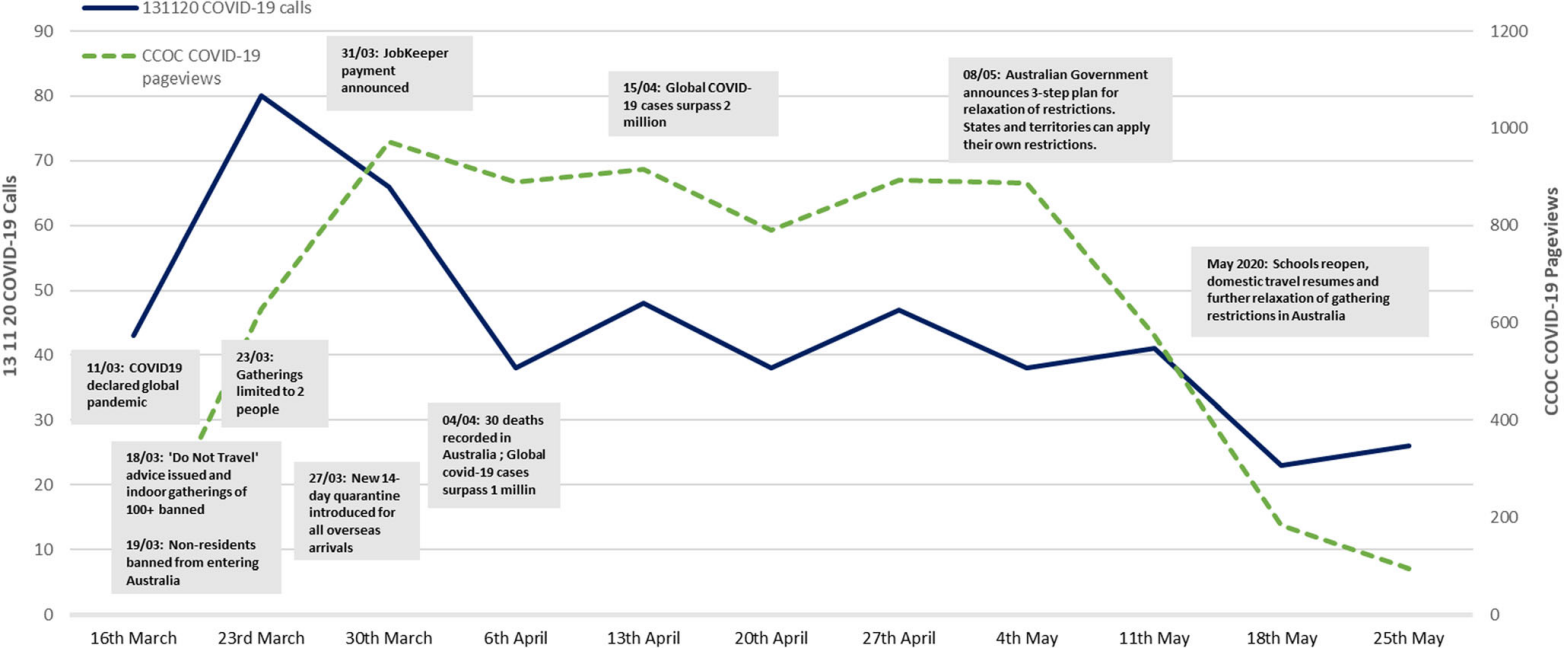
Trends in 131120 call data and CCOC pageviews with key dates of COVID-19 restrictions in Australia. ^*^Due to the low call volumes and online posts received before mid-March 2020, the figure represents data from mid-March 2020–May 2020 to illustrate the key trends

The volume of 131120 calls to CCNSW regarding COVID-19 and pageviews on the CCOC COVID-19-related discussion pages peaked in late March 2020, as the Australian Government began initiating virus control measures from its Emergency Response Plan for Novel Coronavirus 2020 [4] (Fig. 2). These calls and pageviews progressively tapered throughout April and May 2020. The ‘reason for call’ data recorded by 131120 consultants indicated that queries about general COVID-19 information significantly decreased between March and May 2020 (20 to 2%), whereas the proportion of calls regarding psychological distress and emotional support increased from 31% in March 2020 to over 40% in April and May 2020 (Supplementary Table 1).

### Distress levels

The distress thermometer data revealed high levels of distress for people affected by cancer during the pandemic (Supplementary Table 2). During the last 2 weeks of May 2020, average (median) self-reported distress levels on COVID-19-related calls peaked at 8 out of 10: the highest levels observed over the study period and indicative of high distress. Comparatively, average distress reported on COVID-19-related calls between 01 December 2019–31 May 2020 (7/10) was higher than observed for all 131120 calls received in the same time period and in an equivalent time period during the previous year (6/10).

### Qualitative analysis

Qualitative content analyses resulted in the emergence of five dominant themes associated with COVID-19 that were consistent across both the 131120 call data and CCOC posts: (1) psychological distress and fear of virus susceptibility, (2) practical issues, (3) disruptions to cancer treatment and services, (4) information needs, and (5) carer-specific issues. These were then further categorised into various sub-themes. Frequencies of a given theme from the 131120 call and CCOC post data are shown in Table 2, along with illustrative quotes.

**Table 2:** Major themes, sub-themes, and frequencies of qualitative data from 13 11 20 calls and CCOC online posts supported by illustrative quotes*

	131120	CCOC		
Theme	Frequency of main themes (% of *N*)		Sub-themes	Illustrative quotes*
Psychological distress and fear over virus susceptibility	53%	33%	Anxiety about perceived susceptibility	“I would be lying if I said I wasn't worried about my heightened risk.” “I finished my breast cancer treatment a few months ago, after almost a year of chemo and radiotherapy. I'm really worried about whether I am immune-supressed and my risk of getting COVID.”
			Psychological distress	“I have a really good support system. COVID makes it hard though. Lately, the anxiety is eating me, I feel no one understands me and I just vent at my husband. Logically, I'm grateful to everyone but there's this side of me that is negative, isolated and just trying to get through the day.” “I had found ways to cope with cancer but I'm really struggling with everything now with the extra worry of COVID19. I don't feel like anyone understands what the fear is like”
			Feelings of isolation	*“*I feel totally alone. I could cope with the cancer or COVID19, but not both together. It's too much.” “I feel so alone even though [I] have support. The COVID pandemic has left me feeling even more alone because every time I see a good mate we can't hug and keep our distance. I understand it totally, but I really need a hug. I'm trying to be brave.” “I'm feeling really isolated at the moment as I have advanced cancer and haven't been able to leave the house. All the usual social activities and support that I get have gone due to the COVID restrictions.”
Practical issues	33%	9%	Employment issues	I'm really worried about going back to work at this end of this month because of COVID-19. I finished my cancer treatment recently and I'm not technically in remission. I’ve been told that I am immune supressed so I think going back to work might put me at risk so I don’t know what to do? “I'm worried about COVID-19 at the moment. I had cancer a few years ago and received treatment that has affected by immune system. I work as a teacher so I'm really worried about being exposed to the virus. It makes me feel constantly on edge.”
			Financial concerns	“I’ve had some complications following my recent cancer surgery and need to have another scan. My surgeon is insisting that I need it but I can’t afford it at the moment as my partner’s business is really struggling because of COVID-19.” “I have cancer and young children so things have been really difficult for me at the moment. My husband recently lost his job due to COVID19 and it’s made me really worried and upset about my ability to provide for them.”
			Access to essential items	“I have recently finished my chemotherapy and don't have anyone to do my shopping for me. I went to the supermarket this morning and couldn't get any of the things I needed because all the shelves were empty. I'm so stressed that I'm having to put myself at risk of COVID-19 by going out in public and I can't even buy toilet paper.”
			Transportation	“...I have to travel 40 minutes away for a biopsy. I have no reliable transportation at this time, and with the coronavirus, It won't be scheduled for quite some time. I live in a very rural area... on a fixed income and can't afford to pay someone to take me. I also have no family. I called social services too but had no luck...” “I need to get to the hospital every few weeks for my chemotherapy but I'm worried about taking public transport due to COVID-19. My doctor has also told me to avoid public transport too but I don't drive so I'm not sure what to do. I have been taking taxi's recently but it's really expensive and I won't be able to keep doing this.”
Disruptions to cancer treatment and services	11%	26%	Delay or cancellation of appointments or treatment	“I was diagnosed with cancer a few weeks ago but got told yesterday that due to COVID risk my surgery has been delayed. I feel really isolated and worried about the delays and what this means for the cancer.” “My GP recently referred me for a mammogram as I found a lump but all BreastScreen services have been cancelled because of COVID19. I'm really worried it might be cancer”
			Self-elected cancellations	“I've noticed some changes to my skin recently that I probably need to see GP about but I'm putting it off as I'm really worried about going there because of COVID-19.” “I want to cancel my hospital appointment as don’t think it’s worth the risk at the moment with COVID-19”
			Transition to telehealth services	“I have just had a phone call from the outpatient clinic, the doctors have cancelled all appointments tomorrow, they don’t want people coming in, I will receive a text setting up a phone consult with my Surgeon.” “I’ve been referred to a psychologist at the hospital as I've been struggling with things lately due to my cancer diagnosis. I've managed to get a telephone appointment now but I'm really disappointed not to be able to see someone face to face.”
			Concerns of cancer growth and long-term prognosis	“I am currently off my chemo...I can already feel pain & growth in some places where I've had [metastatic disease] before. Hopefully, I'll be able to go back on it sooner rather than later. Life goes on, we must suck it up as best we can”
Information needs	7%	44%	Lack of clear and consistent information about virus susceptibility	“I would really appreciate it if someone could inform those of us who are currently undergoing or have recently completed chemotherapy how compromised our immune systems are in relation to Covid-19. There seems to be no information on the web - except a general comment that those with cancer are at greater risk. That's a massive umbrella statement which gives no useful information.”
			Uncertainty around recommended safeguarding measures	“There isn’t any information, from what I have seen, on what people with chronic disease and low immune systems do to navigate transport, hospitals, medical clinics or even supermarkets safely.”
			Absence of consistent information about the use of personal protective equipment	“I'm just wondering about masks. There's a lot of conflicting information about wearing masks. Globally, 50 doctors have died from the virus and I don't know how many nurses. That's really frightening. If professionals cannot protect themselves, I worry that we [won’t] be able to.” “What is our right [as patients]? Can we ask the doctors, nurses and other medical staff to wear facemasks? I am thinking of writing a letter to my specialists and the management of the hospital.”
Carer-specific issues	20%	11%	Concerns around not being able to provide emotional & practical support	“I feel alone because family and friends can't help out and most support services are closed down.”
			Visitation issues	“I haven't been able to get in there to [visit] him [in hospital]. He doesn't want me to risk getting the virus. He is in a lot of pain though.” “My partner is dying in hospital and I'm so distressed that I might not be there when he passes away. COVID has meant that I can only visit him at certain times.” My mum is receiving palliative care and she's been deteriorating. She will probably be moved into hospital shortly and I'm devastated that I might not get the chance to see her before she dies because of COVID restrictions.”
			Burn out	“It[s] so tiring and stressful dealing with it all on top of coronavirus. I am doing everything for my parents as well as trying to look after myself... and as a single parent [with my own family] ... I’m trying to stay positive & happy but have my times when it’s all too much.”
			Concerns about being an exposure risk to others	“My wife was diagnosed via test as positive yesterday for COVID19…. so, chances are very high I also have it (no symptoms though). My wife is currently in quarantine.” “My husband has cancer and I'm concerned about bringing the virus home with me from school as I'm a teacher. I'm taking all the necessary precautions, but I am constantly worried.”

*Identifiable information has been modified to protect the confidentiality of service user, as per study ethics requirements

**Some of the quotes have also been used in the main text to emphasise key themes

#### Psychological distress and fear of virus susceptibility

People affected by cancer (patients, survivors, and carers) expressed significant psychological distress and fear associated with COVID-19, a theme mentioned in more than half (53%) of the 131120 calls analysed and a third (33%) of the CCOC posts. For many cancer patients and survivors, considerable anxiety was reported around their perceived susceptibility to COVID-19 due to immuno-suppressant effects of active or previous cancer treatment(s) (e.g. chemotherapy, radiotherapy, or medications).

I finished my breast cancer treatment a few months ago, after almost a year of chemo and radiotherapy. I'm really worried about whether I am immune-supressed and my risk of getting COVID.-131120 caller

Feelings of isolation, boredom, and a lack of social support were commonly described due to the impact of social distancing measures and restrictions on travel or movement. Symptoms consistent with depression and other mental health issues were often identified including insomnia, persistent low mood and tearfulness, and a loss of interest in social activities [20].

I'm feeling really isolated at the moment as I have advanced cancer and haven't been able to leave the house. All the usual social activities and support that I get have gone due to the COVID restrictions.-131120 caller

#### Practical issues

Practical issues experienced by people affected by cancer throughout the pandemic included deepening financial hardship, employment changes, and loss of work/household income due to the wide-reaching economic impacts of the crisis. Callers and online posters faced challenging decisions around whether to continue working, especially if employed in essential services that may have an increased risk of exposure, such as hospitals and schools.

I'm really worried about going back to work at this end of this month because of COVID-19. I finished my cancer treatment recently and I'm not technically in remission. I’ve been told that I am immune supressed so I think going back to work might put me at risk so I don’t know what to do?-131120 caller

As a vulnerable population, many following guidance to self-isolate consequentially faced significant challenges in obtaining essential items such as groceries and prescription medications. Temporary item shortages and difficulties accessing online services heightened distress levels for those affected by cancer, particularly where they could not access protective equipment (i.e. face masks) or online services were the only option available to prevent physical contact. Additionally, online delivery was not always possible for those with limited internet access or who resided in a remote area.

I have recently finished my chemotherapy and don't have anyone to do my shopping for me. I went to the supermarket this morning and couldn't get any of the things I needed because all the shelves were empty. I'm so stressed that I'm having to put myself at risk of COVID-19 by going out in public and I can't even buy toilet paper.-131120 caller

Transportation was also another significant practical issue identified, arising from fears of taking public transport and the cancellation of many community transportation services in response to COVID-19. In some cases, transport disruptions resulted in patients being unable to access cancer treatments and medical appointments.

I need to get to the hospital every few weeks for my chemotherapy but I'm worried about taking public transport due to COVID-19. My doctor has also told me to avoid public transport too but I don't drive so I'm not sure what to do. I have been taking taxi's recently but it's really expensive and I won't be able to keep doing this.-131120 caller

#### Disruptions to cancer treatment and services

Both cancer patients and carers highlighted major disruptions to their care and treatment due to COVID-19. Uncertainties were identified around the continuation of treatment, delays to scheduled surgeries, and the availability of hospital resources. They commonly described instances where existing appointments had been rescheduled, cancelled, or changed to a telehealth consultation, in some cases without clear explanation.

I was diagnosed with cancer a few weeks ago but got told yesterday that due to COVID risk my surgery has been delayed. I feel really isolated and worried about the delays and what this means for the cancer. -131120 caller

Such disruptions caused fear around disease progression, and uncertainty about whether the virtual delivery of healthcare would compromise the quality of care they received. It was also evident that in some instances, individuals were self-electing to postpone or cancel appointments due to fears of attending treatment centres during the outbreak.

I've noticed some changes to my skin recently that I probably need to see GP about but I'm putting it off as I'm really worried about going there because of COVID-19.-131120 caller

#### Information needs

Information needs were identified in 44% of CCOC posts, although were less prevalent in the 131120 call notes (7%) (Table 3). The content analysis indicated a lack of clear and consistent information and guidelines for people with a current or previous cancer diagnosis. Several queries were raised both in relation to the risk of COVID-19 infection and whether immunosuppression was a risk factor.

I would really appreciate it if someone could inform those of us who are currently undergoing or have recently completed chemotherapy how compromised our immune systems are in relation to Covid-19. There seems to be no information on the web - except a general comment that those with cancer are at greater risk. That's a massive umbrella statement which gives no useful information.-CCOC online post

**Table 3:** Recommendations to guide needs-based interventions for Australian cancer care during COVID-19

Recommendation theme	Recommendation	Description
Support	Increased psychological support	Greater focus is needed on developing targeted psychological support interventions that specifically meet the needs of people affected by cancer in the context of COVID-19. Interventions should address the distinct set of emotional challenges this pandemic poses for cancer patients, survivors, and their carers, noting that mental health interventions developed for the general population may be less well suited to the specific needs of vulnerable groups [28].
	Effective communication regarding changes to cancer care plans	Changes or modifications to cancer treatment plans should be discussed with patients and carers within the framework of shared decision making. They should be fully informed about the rationale behind unanticipated changes to their cancer care plan, in addition to the risks and benefits of such alterations. Clinicians and health workers involved in cancer services may require further support and training to deliver this information and support effectively.
Access	New models of telehealth-based supportive care	In the absence of face-to-face contact and physical connection, alternative and novel supportive care options need to be developed that ensure safe contact. Where appropriate, secure digital platforms may provide opportunities to improve social connectivity and peer support where face-to-face interactions are not possible. Telehealth may have particular value in the context of cancer survivorship, where the focus is less on active cancer surveillance and more on the transition to normal life [29]. The important role of carers also needs to be considered in the context of telehealth to ensure they have opportunities to be part of care and treatment discussions. New digital models of care should be co-designed and tested with end-users alongside implementation to ensure its appropriateness and acceptability. Further, comprehensive training and support for healthcare professionals is needed to effectively manage transitions to new care modalities.
	Visitation flexibility on compassionate grounds	Patients placed in isolation for clinical reasons may be at increased risk of experiencing depression, anxiety, anger, and loss of self-esteem; which has implications for patient safety [30]. Flexible protocols with respect to visitation for close relatives at cancer treatment centres and hospices should be explored, in consideration of the appropriate virus protection measures. This may have particular value in those circumstances which pose increased risk of high psychological distress for patients, carers and family members (e.g. end of life care, border restrictions, intensive treatment support).
Information	Timely information and guidance	As COVID-19 evidence evolves, information resources and guidance should be continuously updated to ensure people affected by cancer are equipped with timely and reliable information. Information should attempt to address specific barriers and gaps in knowledge such as the impacts of certain cancer treatments and how to best minimise the risk of infection. Such information should be pitched in a way that is accessible for those with low literacy. Information repositories have recently emerged in Australia [31], containing evidence-based resources and guidance, however, these may require further promotion and regular updates accordingly. Targeted information resources also need to be considered specifically for underserved populations such as CALD and Aboriginal and Torres Strait Islander communities.
	Improved communication of virus control measures operationalised in healthcare settings	Reassurance should be provided to both the general population and to cancer patients regarding safety measures and operational changes within Australian healthcare settings. This information may prevent fear-based decision making that could ultimately delay diagnosis and treatment. Targeted public health campaigns encouraging the public to attend regular cancer screening appointments may also be necessary to prevent the late detection of cancer and subsequent adverse clinical outcomes.

Ambiguous sentiment was also apparent in relation to the precautionary measures they should be taking to reduce their risk of virus exposure. They were often uncertain about the extent to which they should be self-isolating at home and required information regarding protective equipment they should wear in public (e.g. masks). Carers sought similar information and advice about how to best safeguard their loved ones and prevent potential exposure to the virus, whilst balancing their responsibilities for providing essential care and emotional support.

#### Carer-specific issues

Carers of people affected by cancer described substantial challenges throughout COVID-19. Restrictions on healthcare centre visitations and travel have limited opportunities for social connection with loved ones, leaving them unable to provide the same levels of practical and emotional support. They frequently expressed anguish that relatives were isolated with limited familial support for prolonged periods, particularly where the cancer was advanced, or they were receiving end of life care.

My partner is dying in hospital and I'm so distressed that I might not be there when he passes away. COVID has meant that I can only visit him at certain times.- 131120 caller

Carers were also concerned about the risks of ‘bringing the virus home’, especially if they were unable to work remotely if employed in essential services such as hospitals, supermarkets, and schools. Challenges were also identified regarding visa arrangements and treatment options for family members visiting Australia from overseas, who were unable to return to their home country due to travel restrictions.

My husband has cancer and I'm concerned about bringing the virus home with me from school as I'm a teacher. I'm taking all the necessary precautions, but I am constantly worried. - 131120 caller

## Discussion

This combination of real-time descriptive data and qualitative insights from existing cancer support services is an Australian-first and demonstrates the significant and multifaceted psychosocial impacts that COVID-19 presents to those affected by cancer. Our five identified themes were not mutually exclusive, and several instances of overlap were evident. For example, unmet information needs—including a lack of understanding around virus susceptibility, and uncertainty around recommended safeguarding measures—were often precursors to expressions of distress and fear.

The long-term repercussions of the pandemic remain unclear, however, our highlight that some patients are self-electing to postpone or cancel their cancer appointments and treatment, corroborating with earlier evidence illustrating declining incidence rates and referrals [11]. As such, the downstream health system effects of delayed diagnoses and possible disease progression may continue to emerge.

Furthermore, in our study, the highest self-reported levels of distress were observed during the last two weeks of May 2020, yet distress levels may have continued to grow or remain high following our study period. In late June 2020, the state of Victoria announced a state-wide lockdown due to exponentially rising cases of community transmission of coronavirus that lasted for over 110 days [21]. In addition, several other virus clusters have since emerged in Australia, affecting NSW, South Australia, Queensland, and Western Australia. The unpredictable nature of the virus poses a long-term public health challenge and ongoing psychological impacts for the population. As such, ongoing research regarding the mental health repercussions of COVID-19 will be important.

In comparison to other countries, Australia’s swift public health action has limited cases of community transmission and associated mortalities [22]. Despite this more favourable position, the ongoing uncertainty, disruption, and social isolation associated with unpredictable lockdowns and state border closures may contribute to lingering psychological distress. A ‘call to action’ has been advocated for cancer programs to actively engage patients with cancer through a transparent flow of communication and prioritisation of psychosocial needs [23] to mitigate some of these issues.

Our study provides a unique contribution to the literature as it is one of the few to consider carer perspectives of cancer care during the pandemic. Carers reported challenges with obtaining information regarding the virus and issues with restricted visitation rights, especially for those in palliative care. Shalowitz et al. discuss considerations around ethical principles and the need for critical communication associated with cancer care, and further suggest disruptive changes and efforts to reduce community transmission of coronavirus cases have devalued the benefits of familial/carer support to cancer patients by restricting access and visitation [24]. While mitigating the risk of virus transmission in high-risk settings is of the utmost importance, innovative strategies that allow for enhanced connection and communication such as telehealth/videoconferencing and visitation on compassionate grounds require further exploration. Since the start of the pandemic, there have been innovations taken forward that relate to our recommended priority areas. For example, throughout 2020, the Australian Government has introduced new temporary Medicare Benefits Schedule (MBS) telehealth items [25] in a bid to protect patients and frontline health workers from the risk of virus exposure. However, a long-term strategic vision for the future implementation of telehealth services in cancer settings is yet to materialise. Furthermore, Cancer Australia released a report detailing a conceptual framework for the management of cancer patients during a pandemic [26], a testament toward efforts for improved communication and information for both patients and healthcare professionals. However, gaps still remain and further work is required to provide appropriate and effective resources to patients with cancer and their carers.

With this and other challenges associated with the pandemic in mind, this study provides an evidence-base for six recommendations that align with these priorities and address support, access, and information needs, offering flexible and adaptable strategies for cancer care throughout the pandemic (Table 3). These include (1) increased psychological support, (2) improved communication of changes to cancer care plans, (3) utilising new models of telehealth-based supportive care, (4) consideration of visitation flexibility on compassionate grounds, (5) provision of timely information and guidance, and (6) improved communication of virus control measures operationalised in healthcare settings.

## Strengths and limitations

These findings should be considered in light of the study limitations. Collected data are from a convenience sample and likely to reflect the needs of individuals who required assistance from specific CCNSW services (e.g. financial or legal assistance). Therefore, our sample may be over-representative of people who have proactively sought help from cancer support services and hence distress levels could be heightened when compared to the wider population of those affected by cancer. Additionally, whilst the search terms were comprehensive, certain spelling variations related to the pandemic or other uncommon terms may not be accounted for within extracted data. Finally, as the pandemic and associated psychosocial impacts continuously evolve, and new government restrictions and policies may follow, the opportunity for ongoing assessment is primed.

## Conclusion

The COVID-19 pandemic is having a profound impact on the emotional wellbeing of cancer patients, survivors, and their carers. The perceived lack of clear guidance, significant disruptions to the delivery of cancer care, and deepening economic and social impacts of the pandemic are further exacerbating these mental health impacts.

These disruptions are likely to be enduring and may have lasting consequences, directly and indirectly impacting on clinical and quality of life outcomes [27]. Monitoring of the lived experiences of those affected by cancer is critical for effective and timely management of supportive care needs of people with cancer during and beyond the COVID-19 crisis. While this study reflects on real-time issues identified from individuals assessing supportive care, there are several considerations around psychological distress and quality of clinical care cancer that warrant further investigation. Future research should focus on the development and implementation of innovations to ensure psychosocial care is considered during crisis events such as pandemics.

## Supplementary information

ESM 1 (PDF 328 kb)

## Data Availability

The 131120 dataset that was extracted and analysed during the current study is not publicly available due to the confidential nature of the service and conditions imposed during ethics approval. Data analysed from the CCOC are publicly available at: [https://onlinecommunity.cancercouncil.com.au/t5/Cancer-and-COVID-19/bd-p/cancercovid19].
