# Supplementary material for: Psychosocial impact of COVID-19 on cancer patients, survivors, and carers in Australia: a real-time assessment of cancer support services
Source: Support Care Cancer. 2021 Mar 11;29(9):5463–73. doi: 10.1007/s00520-021-06101-3 (PMC7946616; doi:10.1007/s00520-021-06101-3)
Supplement: Supplementary file 1 — (PDF 328 kb) [file 520_2021_6101_MOESM1_ESM.pdf]

Psychosocial impact of COVID-19 on cancer patients, survivors and carers in Australia: a real-time assessment of cancer support services

Supplementary Material

Supplementary Table 1: Main Reason for Call recorded by 131120 Consultant for COVID-19 related calls made to 131120 during the study period

Reason for call	March 2020* (n=159)	April 2020 (n=195)	May 2020 (n=141)
Psychological/Emotional Support	31%	43%	40%
Practical Issues	23%	32%	33%
Treatment and Management	14%	9%	9%
General Information	20%	6%	2%
Early Detection/Screening/Symptoms	3%	5%	7%
Diagnosis	4%	4%	7%
Prevention/Risk Factors	4%	1%	1%
Recurrence/Progression of Disease	1%	2%	1%
Total	100%	100%	100%

** before March 2020 only 1 call was recorded relating to COVID-19*

Supplementary Table 2: Weekly average self-reported distress levels for 131120 callers that discussed COVID-19 between 01 December 2019 and 31 May 2020

Week Commencing	Total Number of 131120 Calls	Average (Median) Self-Reported Distress Level for COVID-19 related calls (0-10 scale)	Average (Mean) Self-Reported Distress Level for COVID-19 related calls (0-10 scale) / Standard Deviation
01 Dec 19 -01 Mar 2020	1	NA*	NA*
02-Mar-20	3	NA*	NA*
09-Mar-20	4	NA*	NA*
16-Mar-20	43	5.0	5.9 (3.3)
23-Mar-20	80	7.0	6.7 (2.7)
30-Mar-20	66	7.0	7.0 (2.1)
06-Apr-20	38	7.0	6.9 (2.6)
13-Apr-20	48	6.5	6.1 (2.4)
20-Apr-20	38	7.0	6.5 (2.9)
27-Apr-20	47	6.0	5.8 (2.4)
04-May-20	38	8.0	7.2 (1.9)
11-May-20	41	5.0	5.8 (3.1)
18-May-20	23	8.0	7.5 (0.9)
25-May-20	26	8.0	7.5 (1.8)
**Total / Overall Average**	**496**	**7.0**	**6.6 (2.3)**

*NA - Prior to the week commencing 16th March 2020, COVID-19 related call volumes on 13 11 20 were too low for a reliable average calculation (01 Dec 2019 – 15 March 2020 n=8

Supplementary Table 3: Methods Used to Ensure Rigour in Qualitative Analysis

Criteria for Rigour	Method of Ensuring Rigour
**Credibility**	Rich excerpts from the transcripts were provided in text to ground the interpretations<br>The researchers searched for instances of inconsistency within categories and omitted codes/categories with little evidence<br>Data analysis procedures were reviewed iteratively by the research team to prevent bias and selective inattention
**Fittingness**	Typicality of participants from 131120 was established by collecting demographic data from national minimum database.<br>The sampling resulted in a range of participants who represented both cancer patients and carers.<br>The study results fit the data from which they were generated as demonstrated by quotes appearing in the results section and in Table 3.
**Auditability**	Call transcripts from 131120 are stored at CCNSW. Online posts made to the CCOC are publicly available.<br>The researchers kept notes as to developing codes and themes to ensure accurate recall<br>Descriptions of the strategies used to collect and analyse the data have been detailed in text<br>Characteristics of participants from 131120 calls are described in Table 1<br>The strategies used to sample calls and online posts have been described in text<br>Twenty percent of the data was collaboratively coded with the researcher's supervisors to ensure identification of the same codes and categories

Supplementary Table 4: Standards for Reporting Qualitative Research (SRQR)*

http://www.equator-network.org/reporting-guidelines/srqr/

Title and abstract

	**Page(s).**
**Title** - Concise description of the nature and topic of the study Identifying the study as qualitative or indicating the approach (e.g., ethnography, grounded theory) or data collection methods (e.g., interview, focus group) is recommended	Page 1
**Abstract** - Summary of key elements of the study using the abstract format of the intended publication; typically includes background, purpose, methods, results, and conclusions	Page 2

Introduction

**Problem formulation** - Description and significance of the problem/phenomenon studied; review of relevant theory and empirical work; problem statement	Page 4
**Purpose or research question** - Purpose of the study and specific objectives or questions	Abstract and Page 4

Methods

**Qualitative approach and research paradigm** - Qualitative approach (e.g., ethnography, grounded theory, case study, phenomenology, narrative research) and guiding theory if appropriate; identifying the research paradigm (e.g., postpositivist, constructivist/ interpretivist) is also recommended; rationale**	Methods Pages 4-6
**Researcher characteristics and reflexivity** - Researchers' characteristics that may influence the research, including personal attributes, qualifications/experience, relationship with participants, assumptions, and/or presuppositions; potential or actual interaction between researchers' characteristics and the research questions, approach, methods, results, and/or transferability	Supplementary table 3
**Context** - Setting/site and salient contextual factors; rationale**	Methods Pages 4-6
**Sampling strategy** - How and why research participants, documents, or events were selected; criteria for deciding when no further sampling was necessary (e.g., sampling saturation); rationale**	Methods, 'Sampling' page 4
**Ethical issues pertaining to human subjects** - Documentation of approval by an appropriate ethics review board and participant consent, or explanation for lack thereof; other confidentiality and data security issues	Declarations page 13
**Data collection methods** - Types of data collected; details of data collection procedures including (as appropriate) start and stop dates of data collection and analysis, iterative process, triangulation of sources/methods, and modification of procedures in response to evolving study findings; rationale**	Methods Pages 4-6

**Data collection instruments and technologies** - Description of instruments (e.g., interview guides, questionnaires) and devices (e.g., audio recorders) used for data collection; if/how the instrument(s) changed over the course of the study	Methods Pages 4-6
**Units of study** - Number and relevant characteristics of participants, documents, or events included in the study; level of participation (could be reported in results)	Results, page 6
**Data processing** - Methods for processing data prior to and during analysis, including transcription, data entry, data management and security, verification of data integrity, data coding, and anonymization/de-identification of excerpts	Methods, 'data analysis' page 5
**Data analysis** - Process by which inferences, themes, etc., were identified and developed, including the researchers involved in data analysis; usually references a specific paradigm or approach; rationale**	Methods, 'data analysis' page 5
**Techniques to enhance trustworthiness** - Techniques to enhance trustworthiness and credibility of data analysis (e.g., member checking, audit trail, triangulation); rationale**	Supplementary table 4

Results/findings

**Synthesis and interpretation** - Main findings (e.g., interpretations, inferences, and themes); might include development of a theory or model, or integration with prior research or theory	Page 6-11
**Links to empirical data** - Evidence (e.g., quotes, field notes, text excerpts, photographs) to substantiate analytic findings	Page 6-11

Discussion

**Integration with prior work, implications, transferability, and contribution(s) to the field** - Short summary of main findings; explanation of how findings and conclusions connect to, support, elaborate on, or challenge conclusions of earlier scholarship; discussion of scope of application/generalizability; identification of unique contribution(s) to scholarship in a discipline or field	Discussion page 12
**Limitations** - Trustworthiness and limitations of findings	Discussion page 12

Other

**Conflicts of interest** - Potential sources of influence or perceived influence on study conduct and conclusions; how these were managed	Declarations page 13
**Funding** - Sources of funding and other support; role of funders in data collection, interpretation, and reporting	Compliance with ethics standards, 'Funding' page 14

*The authors created the SRQR by searching the literature to identify guidelines, reporting standards, and critical appraisal criteria for qualitative research; reviewing the reference lists of retrieved sources; and contacting experts to gain feedback. The SRQR aims to improve the transparency of all aspects of qualitative research by providing clear standards for reporting qualitative research.

**The rationale should briefly discuss the justification for choosing that theory, approach, method, or technique rather than other options available, the assumptions and limitations implicit in those choices, and how those choices influence study conclusions and transferability. As appropriate, the rationale for several items might be discussed together.

Reference:
O'Brien BC, Harris IB, Beckman TJ, Reed DA, Cook DA. **Standards for reporting qualitative research: a synthesis of recommendations.** *Academic Medicine*, Vol. 89, No. 9 / Sept 2014
DOI: 10.1097/ACM.0000000000000388
